# A rare face of follicular lymphoma: reverse variant of follicular lymphoma

**DOI:** 10.1186/s13000-020-00932-0

**Published:** 2020-04-03

**Authors:** Ninu Maskey, Qiongrong Chen, Fang Liu, Shangqin Liu, Sufang Tian

**Affiliations:** 1 Department of Hematology, Zhongnan Hospital of Wuhan University, 169 Donghu Road, Wuchang District, Wuhan, Hubei 430071 People’s Republic of China; 2 Department of Pathology, Zhongnan Hospital of Wuhan University, Wuhan University Center for Pathology and Molecular Diagnostics, 169 Donghu Road, Wuchang District, Wuhan, 430071 People’s Republic of China; 3 Department of Pathology, Foshan Hospital, Sun Yat-sen University, 81 North Lingnan Avenue, Chancheng District, Foshan, Guangdong Province People’s Republic of China

**Keywords:** Follicular lymphoma, Centroblasts, Centrocytes, Follicular dendritic cells meshwork, Reverse variant of follicular lymphoma

## Abstract

**Background:**

Reverse Variant of Follicular Lymphoma (RVFL) is one of the rare morphological variants of FL, characterized by dark staining small centrocytes in the center and pale staining large centroblasts at the periphery of the neoplastic follicles. Only rare cases of RVFL have been described to date. The histological appearance of this little known variant of FL may be misinterpreted if pathologists are unaware of its existence. The main purpose of this study is to draw pathologists’ attention to such an uncommon growth pattern of FL so that this variant can be correctly recognized and the clinical significance further studied in the future.

**Methods:**

Four cases of FL with unusual morphologic features were evaluated for the expression pattern of CD20, CD10, BCL6, BCL2, CD21, CD23, CD3, CD5, Cyclin D1, IgD and Ki67 by immunohistochemistry. Fluorescence in situ hybridization (FISH) with break-apart probes was performed to detect *BCL2* gene rearrangement.

**Results:**

All four cases showed distinctive morphologic pattern of RVFL; in addition, each also exemplified unique morphological features. Immunohistochemical stains confirmed the cells in both the central areas and the peripheral cuffs had the same immunophenotypic profiles, contrasting to the FL with marginal zone differentiation in which only the center of the nodules showed expression of CD10. FISH demonstrated *BCL2* gene rearrangement in all cases.

**Conclusion:**

The growth pattern of this rare FL variant may mimic FL with marginal-zone differentiation and other entities including but not limited to marginal zone lymphoma (MZL), progressive transformation of germinal centers (PTGC) and nodular lymphocyte predominant Hodgkin lymphoma (NLPHL). Pathologists should be familiar with this unusual morphological variant to avoid diagnostic pitfalls.

## Background

Follicular lymphoma (FL) is the second most common mature B-cell lymphoma diagnosed in adults in the USA and western Europe [1]. It is characterized by neoplastic follicles arranged in a back-to-back pattern throughout the lymph node with decreased interfollicular area. The follicles are replaced by a proliferation of small centrocytes (small cleaved cells) with angulated nuclei admixed with a variable number of large centroblasts which have more finely dispersed chromatin than the interfollicular small lymphocytes [2]. There are several morphological variants of FL, of which RVFL is one of the little-known and rare faces. RVFL is characterized by neoplastic follicles with pale staining large centroblasts at the periphery and dark staining small centrocytes in the center. Both types of cell in the follicles are neoplastic. A review of the literature found only 3 cases have been reported so far (2 in 1988 and 1 in 2016) [3, 4]. Due to the unusual histologic appearance, RVFL can be misinterpreted by a pathologist unaware of its existence. For this reason, we present four cases of RVFL to illustrate the morphologic and immunophenotypic features. We hope our study can raise awareness of the existence of this unique morphological variant of FL. We also compared the immunostaining pattern of RVFL to FL with marginal-zone differentiation, and discuss the differential diagnoses between RVFL and its morphologic mimics.

## Materials and methods

With approval of the Institutional Review Boards of Wuhan University, Zhongnan Hospital, China, four cases of follicular lymphoma with unusual morphologic features considered to be RVFL were retrieved from the archives of the Department of Pathology, Zhongnan Hospital of Wuhan University. Three cases of FL with marginal-zone differentiation were included for comparison. The features of RVFL at low and high magnification on hematoxylin and eosin (H&E) stained sections were recognized after reviewed by two senior pathologists (Dr. Sufang Tian and Dr. Qiongrong Chen). Formalin fixed paraffin embedded (FFPE) block(s) of each case were then retrieved to perform further ancillary tests for this study. Immunohistochemistry (IHC) was performed using methods published elsewhere [5]. Monoclonal antibody against CD3, CD10, CD21, CD23, BCL2, BCL6, Cyclin D1, Ki67 and anti-IgD polyclonal antibody were obtained from ZSGB-BIO (Beijing, China). Monoclonal antibody against CD5 and CD20 were obtained from LEICA BIOSYSTEMS, China. All the antibodies were purchased in prediluted form.

Fluorescence in situ hybridization (FISH) was performed using 18q21.33 *BCL2* DF probe (Agilent; DAKO, Cedar Creek, USA) to detect *BCL2* gene rearrangement according to the manufacturer’s recommendations and our laboratory developed protocol. Hybridization was performed as mentioned elsewhere [6]. Sections were viewed under a BX60 fluorescence microscope (Olympus, Tokyo, Japan) using a × 100 oil immersion lens and appropriate filters. The result was defined as positive if at least 5% cells show 1 fusion signal (yellow), 1 red signal and 1 green signal.

## Results

### Typical pattern of RVFL with follicles displaying two distinct layers was observed in three cases

**Fig. 1:**
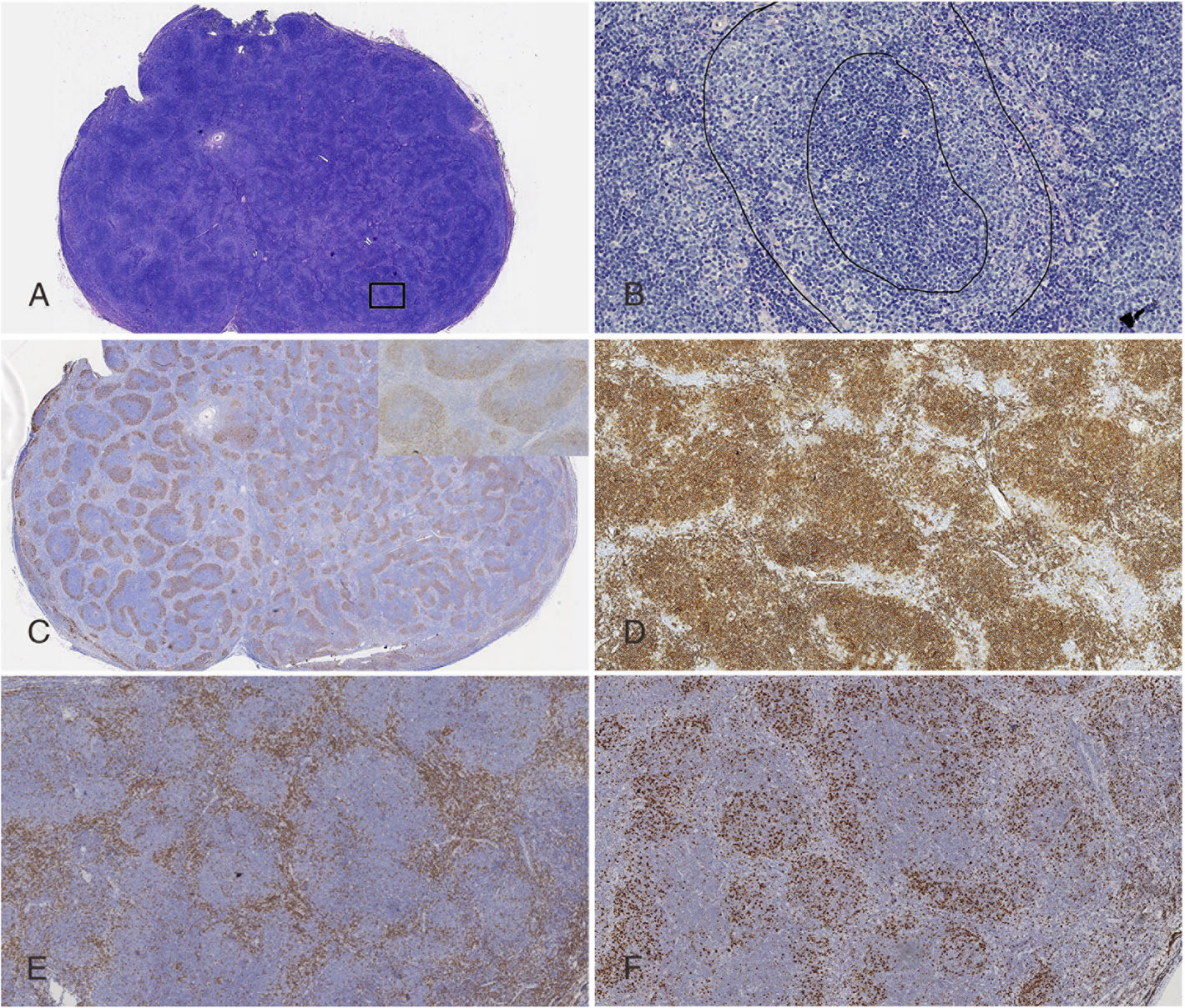
Case 1: **a** and **b** illustrate low-magnification and high-magnification appearances respectively (H&E stain), demonstrating lymph node architecture completely effaced by nodules with dark- staining centers and pale-staining cuffs. The dark- staining center is composed mainly of centrocytes, while the pale-staining cuff contains mainly of centroblasts. IHC staining for CD 21 shows inversely organized follicular dendritic cell (FDC) meshwork at the periphery of the nodules (**c** and high-power inset). The neoplastic cells were immunoreactive with CD10 (**d**) and negative for CD3 (**e**). Ki67 labelling index 40% (**f**)

The first case was a 57-year-old male, presented with generalized lymphadenopathy and splenomegaly. There was also lymphoma involving bone marrow confirmed by biopsy. H&E stained sections of dissected inguinal lymph node demonstrated typical features of RVFL. At low magnification, the lymph node architecture was effaced by variable-sized and closely packed nodules (Fig. 1a). The nodules showed a “reverse pattern” with dark staining centers (mainly composed of centrocytes) surrounded by pale staining cuffs (mainly composed of centroblasts) (Fig. 1b). No obvious tingible body macrophages were identified in the nodules. This case had more than 15 centroblasts per high power field (HPF), so it was diagnosed as FL grade 3A according to the World Health Organization (WHO) classification [7]. IHC staining for CD21 showed follicular dendritic cell (FDC) meshwork inversely organized at the periphery of the nodules (Fig. 1c). The neoplastic cells were strongly postive for CD20 (not shown) and CD10 (Fig. 1d) and negative for CD3 (Fig. 1e). The Ki67 labeling index was approximately 40% (Fig. 1f) in the follicles; and the Ki67 positive cells also highlighted the reversed pattern, i.e. more frequent at the periphery and less in the center.

**Fig. 2:**
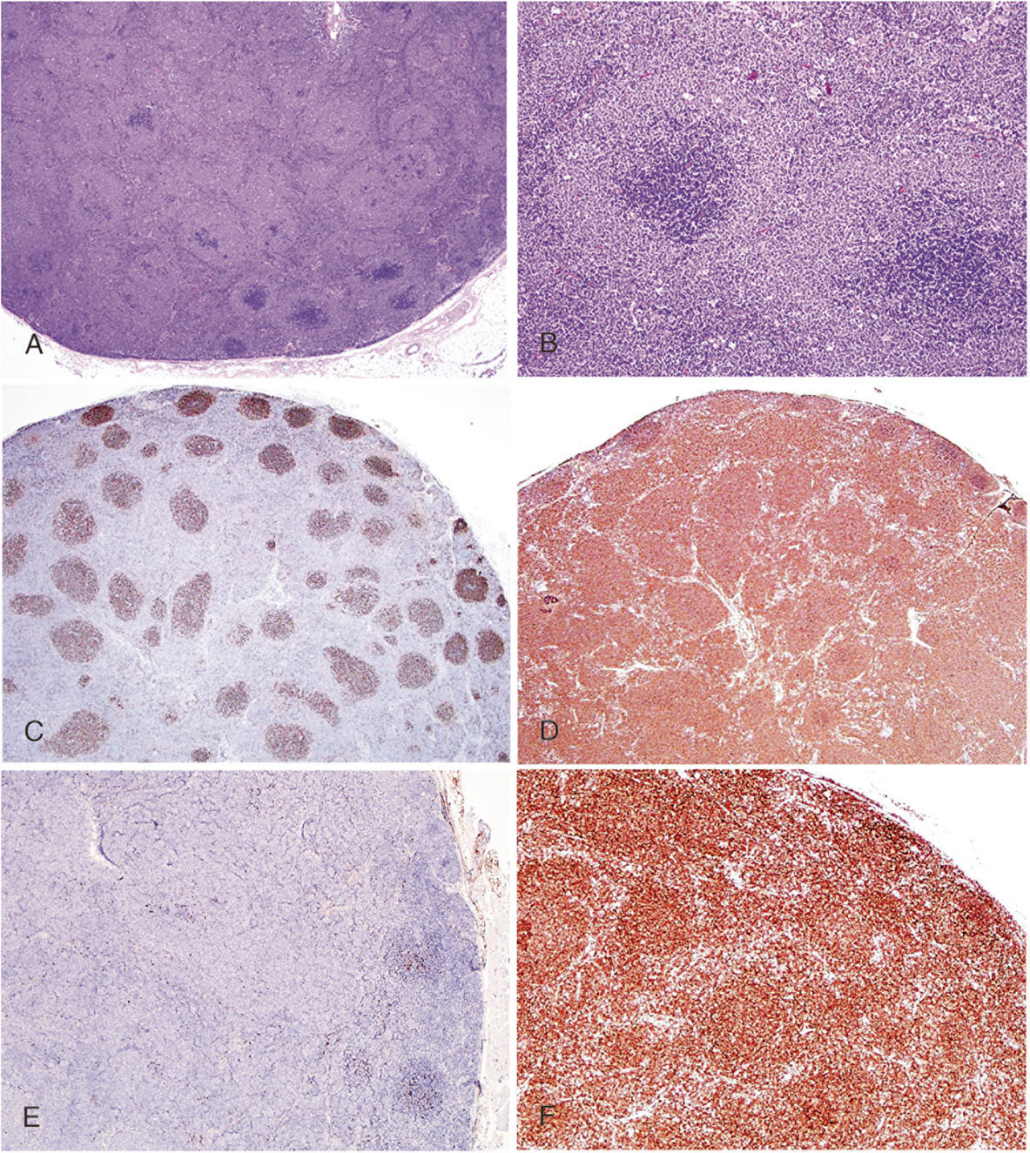
Case 2: **a** and **b** illustrate low-magnification and high-magnification appearance respectively (H&E stain), showing similar morphological pattern as in case 1 except for more expanded pale-staining cuffs. Also note some nodules are devoid of dark areas. IHC staining for CD21 reveals disrupted FDC meshwork (**c**), CD20 and BCL2 staining are positive for neoplastic cells (**d**, **f**) while CD10 staining negative (**e**). The neoplastic cells are also positive for BCL6, HGAL, LMO2 (not shown)

The second case was a 44-year-old male, presented with right cervical mass for a month. The right tonsil was severely enlarged, pushing soft palate towards left. Several enlarged lymph nodes were palpable at the right cervical region. A right cervical lymph node biopsy showed the similar morphological pattern as seen in case 1, except for more expanded pale-staining cuffs. Some neoplastic nodules were devoid of dark area, with the number of centroblasts more than 15 per HPF and focally forming confluent clusters. This case was diagnosed as FL grade 3B (Fig. 2a and b). IHC staining for CD21 revealed disrupted FDC meshwork (Fig. 2c), CD20, BCL6, HGAL, LMO2 and BCL2 stainings were positive in neoplastic cells while CD10 staining was negative (See Fig. 2 D, 2E, and 2F for staining patterns of CD20, CD10 and BCL2).

**Fig. 3:**
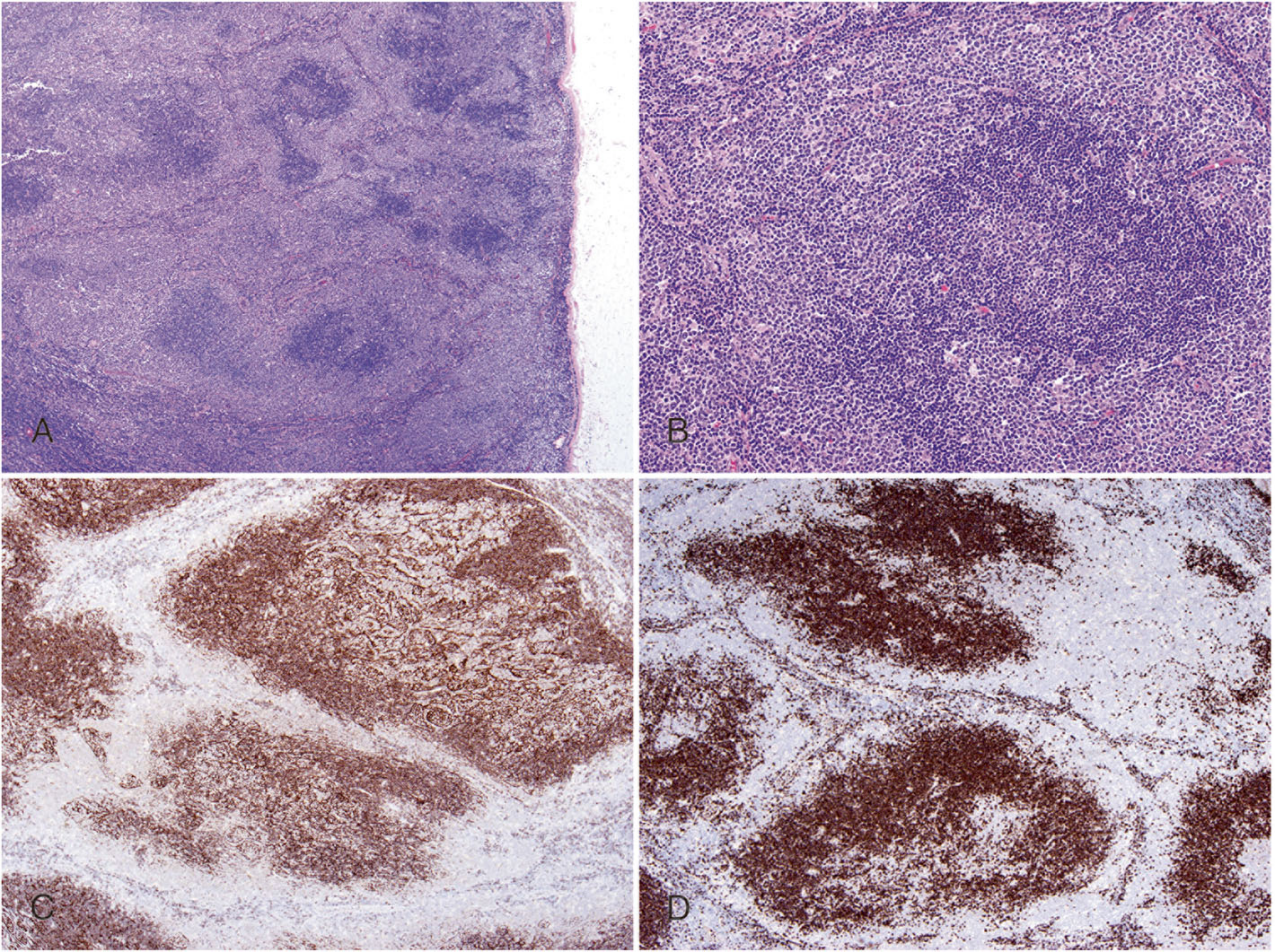
Case 3: **a** and **b** illustrate low-magnification and high-magnification appearance respectively (H&E stain), showing effaced architecture with each follicular unit includes both the dark-staining center and the pale cuff. IHC staining for CD21 shows disorganized FDC meshwork (**c**) and IgD reveals reversed pattern (**d**)

The third case was a previously healthy 50-year-old male, presented with right shoulder mass for a week and was found to have generalized lymphadenopathy. H&E stained sections of the excised cervical lymph node showed effaced architecture with abnormal follicular proliferation. Each follicle displayed both the dark-staining center and the pale cuff (Fig. 3a and b). The FDC meshwork was distorted as shown by CD21 staining (Fig. 3c). The unusual staining pattern of IgD (Fig. 3d) outlining the reversed mantle zone, together with other markers including CD20, CD10, BCL6 and BCL2 (not shown), further supported the diagnosis of RVFL. This case was diagnosed as FL grade 3A.

*BCL2* rearrangement was detected in all three cases by FISH (result not shown).

### Unique pattern of RVFL with follicles displaying three distinct layers was observed in one case

**Fig. 4:**
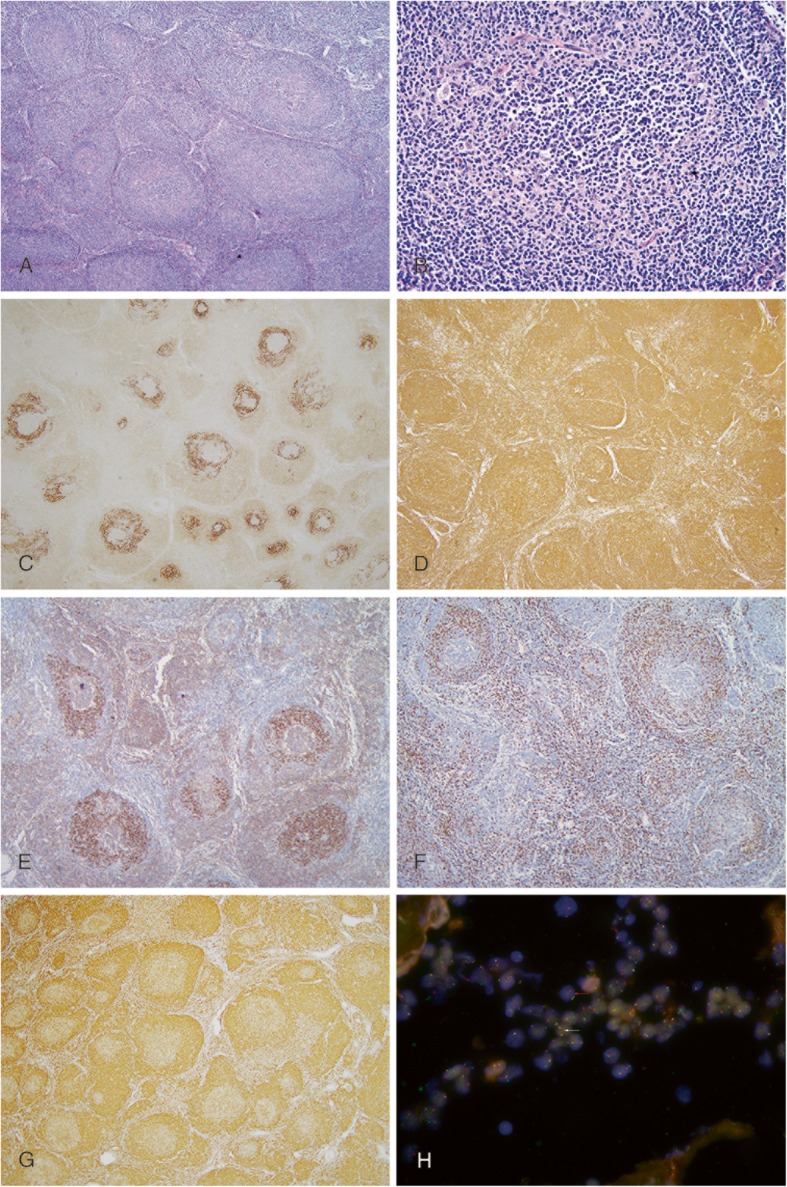
Case 4: **a** and **b** illustrate low-magnification and high-magnification appearance of respectively(H&E stain), showing effacement of the lymph node architecture, replaced by varying sized nodules with the presence of three distinct layers of cells in the nodules comprising inner most layer of dark-staining neoplastic centrocytes surrounded by a layer of pale-staining neoplastic centroblasts followed by the outskirt of a thin layer of small lymphocytes assembled outside the FDC meshwork, confirmed by IHC staining for CD21, CD20, CD10 and BCL2 (**c**,**d**,**e** and **g**). CD3 stain reveals T cells in the outmost layer (**f**). FISH demonstrates rearranged BCL2 gene (**h**), the neoplastic cells show 1- fused, single red, and single green signals (the red arrow) whereas the normal cells show two fused signals (the white arrow)

The fourth case was a 60-year-old female with a history of right lung cancer 2 years ago. On radiologic examination, her abdominal and retroperitoneal lymph nodes were enlarged. Biopsy of a small mesenteric lymph node (maximum diameter 3 cm) rendered the diagnosed of a low grade FL. H&E stained sections of the biopsy displayed slightly different morphological features compared with other three cases described above. Besides effacement of lymph node architecture by the proliferation of variable sized nodules, the unique morphological pattern noted in this case was the presence of three distinct layers of cells in the nodules. The center of each nodule was comprised of dark-staining neoplastic centrocytes, surrounded by a layer of pale-staining neoplastic centroblasts, bordered by a thin outskirt layer of small lymphocytes (Fig. 4a and b). IHC staining for CD21 revealed inversely organized meshwork of FDCs around the centers of the nodules (Fig. 4c), and the outskirt layer was assembled outside the FDCs meshwork. The neoplastic cells were positive for CD20, CD10 and BCL2 (Fig. 4d, e and g). The small lymphocytes in the outermost layer expressed CD3 (Fig. 4f) and CD5 (not shown), indicating that these were actually mature T cells. FISH with break-apart probes showed *BCL2* rearrangement (Fig. 4h).

### RVFL had an immunostaining pattern different from FL with marginal-zone differentiation

**Fig. 5:**
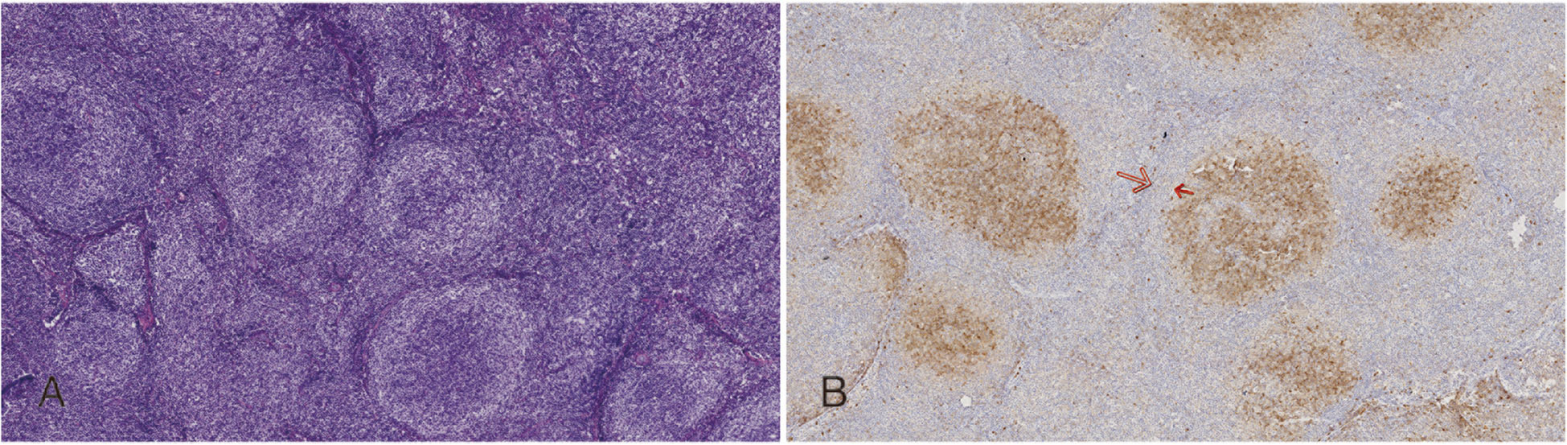
FL with marginal–zone differentiation: H&E stain shows the neoplastic follicles surrounded by broad pale-staining haloes formed by monocytoid B cells. IHC staining for CD10 is positive in the central component, whereas the marginal component is negative (**b**) as shown by the gap between the red arrows

On the H&E stained sections, the lymph nodes of FL with marginal-zone differentiation showed a morphologic pattern similar to RVFL. The neoplastic follicles were surrounded by broad pale-staining zones formed of monocytoid B cells (Fig. 5a). However, in the follicles of FL with marginal-zone differentiation, IHC staining revealed CD10 positivity only in the central area (Fig. 5b), whereas the pale staining marginal zone lacked the follicular center B-cell antigens such as CD10.

## Discussion

FL is regarded as a disease entity with well-defined diagnostic criteria based on morphologic, immunophenotypic and genetic features. Nevertheless, latest discoveries are changing the impression that FL is a disease entity that can be easily diagnosed and classified. Cases of FL with a range of unusual features have been described [8–10]. The morphologic variants of FL were reviewed by Choi and colleagues [11]. Currently recognized variants include FL with marginal zone differentiation, FL with plasmacytic differentiation, FL with Reed-Sternberg-like cells, floral variant, epithelioid variant and signet-ring cell variant. RVFL, which exhibits an unusual morphologic pattern, adds another dimension to the diagnostic challenges. It is of pivotal importance for pathologists to become familiar with this rare face of follicular lymphoma to avoid diagnostic pitfalls.

The 4 cases of FL we presented here differ from the typical FL in that the neoplastic follicles showed a ‘reversed’ pattern on morphologic examination, with each follicle comprised a dark-staining center and a pale-staining cuff. By CD21 staining, the FDC meshwork was present only in the outer cuff of the neoplastic follicles. The B cells in both the centers and the cuffs were neoplastic, since they stained positive for the same markers (CD10, CD20 and BCL2). The diagnosis of FL was further confirmed by a *BCL2* rearrangement demonstrated with FISH in all 4 cases. Among the four cases of RVFL we described, the first three cases showed two distinct layers (biphasic pattern) with dark-staining centrocytes surrounded by pale cuff of centroblasts, whereas, the fourth case showed three layers (triphasic pattern) with the biphasic pattern further bordered by a thin outskirt layer of mature T cells. Although the neoplastic cells still display the biphasic pattern, recognizing the triphasic pattern as demonstrated in our fourth case brings newer insights into the diversity of the morphologic patterns of RVFL.

Considering that the FDC meshwork was patent albeit peripherally distributed in all 4 cases, we speculate that the FDC meshwork is a contributing factor to the unique morphological pattern. Germinal center (GC) dark and light zone organization has been well studied in the animal model by Allen CD and his team who reported the crucial role of chemokine receptors CXCR4 and CXCR5 on the maintenance of the polarity of GC [12]. Whether the chemokine receptors are responsible for the formation of the unique follicular pattern of RVFL may worth further study.

The growth pattern of RVFL can mimic other lymphoproliferative disorders. Several differential diagnoses need to be considered, including but not limited to FL with marginal-zone differentiation, marginal zone lymphoma (MZL), progressive transformation of germinal centers (PTGC), nodular lymphocyte predominant Hodgkin lymphoma (NLPHL) [13–17] and even anaplastic large cell lymphoma. FL with marginal-zone differentiation is one of the rare variants of FL. The characteristic finding in FL with marginal zone differentiation is that the neoplastic follicles have centers surrounded by broad pale-staining haloes formed by monocytoid B cells that have centrocyte-like nuclei but more abundant cytoplasm [11, 17]. The cells in the center express follicular center markers (CD10, BCL6, etc.), whereas the monocytoid cells in the marginal zone lack these antigens (as shown in Fig. 5b). When stained with germinal center B cell markers such as CD10, there is a gap between the outer margin of the neoplastic follicle and the positive staining cells. This is quite different from RVFL where the dark-staining centers and the pale-staining cuffs both demonstrate the same staining pattern for CD10, CD20 and BCL2.

Differentiating RVFL from MZL is not always straightforward. However, in MZL, tumor cells are post-germinal center B cells including monocytoid B-cells, small centrocyte-like cells, plasma cells and transformed B cells. The monocytoid neoplastic cells are monomorphous, medium-sized and possess moderate amounts of pale cytoplasm [18]. The residual lymphoid follicles are reactive, although could be disrupted, and surrounded by a neoplastic cell proliferation that frequently expands into the interfollicular areas. The presence of remnant FDC meshwork suggestive of colonized follicles favors the diagnosis of MZL. Also, the neoplastic lymphocytes in MZL and RVFL show different immunophenotype; the former has no specific markers whereas the latter is usually positive for CD10, BCL6 and other germinal center B cell markers. In addition, these two entities have different genetic profiles that can be tested to distinguish them from each other in challenging cases.

PTGC is a benign condition characterized by enlarged follicles with a nodular proliferation of mantle zone B cells, blurring the defining border between the germinal center and the mantle zone. The nodules may show pale staining cuffs. PTGC differs from RVFL in that the nodules are often large, comprised mainly of small round lymphocytes instead of follicular center cells. Also, it is the clusters of epithelioid histiocytes at the margins of the nodules rendering the pale looking zone of PTGC nodules, whereas the pale cuffs in RVFL mainly comprise centroblasts [14, 19].

The morphological features of NLPHL can appear quite similar to the RVFL at low power view. However, in NLPHL, the nodules are generally ill-defined and larger, consist of predominantly small round lymphocytes and histiocytes. The characteristic cells of NLPHL are LP or popcorn cells (formerly called lymphohistiocytic cells [L&H cells]). When a pale rim is present, it is usually comprised of epithelioid histiocytes instead of centroblasts [16, 20].

FL cases characterized by a plethora of unusual features was reviewed in detail by Xerri and colleague [10]. Although it is unknown whether the reverse pattern in RVFL has any prognostic significance due to limited number of cases studied, our cases add another morphological peculiarity to FL. Of note, due to the peripheral grouping of the large cells in the follicles in RVFL, grading these FL cases could be difficult. Further studies to uncover the molecular basis of different morphologic patterns may contribute valuable information to better diagnostic approach and treatment decisions.

## Conclusion

RVFL is one of the rare faces of follicular lymphoma. The growth pattern of this rare FL variant may mimic FL with marginal-zone differentiation and other entities including but not limited to MZL, PTGC and NLPHL. Recognizing such unusual morphological variant of FL is crucial to avoid diagnostic pitfalls.

## Data Availability

All data for this study are presented in the manuscript.

## Abbreviations

FDC: Follicular dendritic cells
FFPE: Formalin fixed paraffin embedded
FISH: Fluorescence in situ hybridization
FL: Follicular lymphoma
IHC: Immunohistochemistry
MZL: Marginal zone lymphoma
NLPHL: Nodular lymphocyte predominance hodgkin lymphoma
PTGC: Progressive transformation of germinal centres
RVFL: Reverse variant of follicular lymphoma
